# Acid ceramidase is upregulated in AML and represents a novel therapeutic target

**DOI:** 10.18632/oncotarget.13079

**Published:** 2016-11-04

**Authors:** Su-Fern Tan, Xin Liu, Todd E. Fox, Brian M. Barth, Arati Sharma, Stephen D. Turner, Andy Awwad, Alden Dewey, Kenichiro Doi, Barbara Spitzer, Mithun Vinod Shah, Samy A.F. Morad, Dhimant Desai, Shantu Amin, Junjia Zhu, Jason Liao, Jong Yun, Mark Kester, David F. Claxton, Hong-Gang Wang, Myles C. Cabot, Edward H. Schuchman, Ross L. Levine, David J. Feith, Thomas P. Loughran

**Affiliations:** ^1^ Department of Medicine, University of Virginia, Charlottesville, VA, USA; ^2^ Penn State Hershey Cancer Institute, Hershey, PA, USA; ^3^ Department of Pharmacology, University of Virginia, Charlottesville, VA, USA; ^4^ Department of Molecular, Cellular, and Biomedical Sciences, University of New Hampshire, Durham, NH, USA; ^5^ Public Health Sciences, University of Virginia School of Medicine, Charlottesville, VA, USA; ^6^ Department of Pathology, Osaka City University Medical School, Osaka, Japan; ^7^ Human Oncology and Pathogenesis Program and Leukemia Service, Memorial Sloan Kettering Cancer Center, New York, NY, USA; ^8^ Department of Stem Cell Transplantation and Cellular Therapy, University of Texas MD Anderson Cancer Center, Houston, TX, USA; ^9^ Department of Biochemistry and Molecular Biology, East Carolina University, Brody School of Medicine, Greenville, NC, USA; ^10^ Department of Pharmacology, Faculty of Veterinary Medicine, South Valley University, Qena, Egypt; ^11^ Department of Pharmacology, Pennsylvania State University College of Medicine, Hershey, PA, USA; ^12^ Department of Pediatrics, Pennsylvania State University College of Medicine, Hershey, PA, USA; ^13^ Department of Genetics and Genomic Sciences, Icahn School of Medicine at Mt. Sinai, New York, NY, USA; ^14^ University of Virginia Cancer Center, Charlottesville, VA, USA

**Keywords:** acid ceramidase, myeloid cell leukemia sequence 1 protein, ceramide, sphingosine 1-phosphate, leukemia

## Abstract

There is an urgent unmet need for new therapeutics in acute myeloid leukemia (AML) as standard therapy has not changed in the past three decades and outcome remains poor for most patients. Sphingolipid dysregulation through decreased ceramide levels and elevated sphingosine 1-phosphate (S1P) promotes cancer cell growth and survival. Acid ceramidase (AC) catalyzes ceramide breakdown to sphingosine, the precursor for S1P. We report for the first time that AC is required for AML blast survival. Transcriptome analysis and enzymatic assay show that primary AML cells have high levels of AC expression and activity. Treatment of patient samples and cell lines with AC inhibitor LCL204 reduced viability and induced apoptosis. AC overexpression increased the expression of anti-apoptotic Mcl-1, significantly increased S1P and decreased ceramide. Conversely, LCL204 induced ceramide accumulation and decreased Mcl-1 through post-translational mechanisms. LCL204 treatment significantly increased overall survival of C57BL/6 mice engrafted with leukemic C1498 cells and significantly decreased leukemic burden in NSG mice engrafted with primary human AML cells. Collectively, these studies demonstrate that AC plays a critical role in AML survival through regulation of both sphingolipid levels and Mcl-1. We propose that AC warrants further exploration as a novel therapeutic target in AML.

## INTRODUCTION

Acute myeloid leukemia (AML) is a group of heterogeneous hematological diseases [1, 2]. Genetic abnormalities such as chromosomal deletions, inversions and translocations, as well as molecular alterations in hematopoietic stem cells abrogate normal differentiation and induce uncontrolled proliferation [3]. AML patients generally have high levels of these immature leukemic cells, known as blasts, accumulating in the bone marrow and peripheral blood with occasional organ infiltration. Current first-line cytotoxic chemotherapy exhibits limited success, with 50% of younger patients and 80% of older patients succumbing to the disease [2, 4]. Lower survival rates are also observed in patients with prior myelodysplastic syndromes (MDS) and prior chemotherapy treatments [5]. New therapeutics are under development to target specific genetic or molecular abnormalities, but these are limited to selected sub-populations within AML [6]. In this context, it is vitally important to discover novel therapeutic targets for a broader spectrum of AML patients.

Sphingolipids, a class of bioactive molecules, are important in determining cell fate [7]. The balance between two major sphingolipid metabolites: pro-apoptotic ceramide and pro-survival sphingosine 1-phosphate (S1P), has been referred to as the “sphingolipid rheostat” [8]. Studies have shown that endogenous ceramide functions as a pro-apoptotic lipid messenger when induced by radiation, stress, and chemotherapeutic agents [9]. Furthermore, increased ceramide levels trigger apoptotic cell death in several types of cancer [10], either through receptor-mediated caspase activation or through mitochondrial-mediated caspase activation involving cytochrome *c* release [11, 12].

Ceramidases are a group of enzyme hydrolases within the sphingolipid pathway that metabolize ceramide into sphingosine and free fatty acid [10]. Sphingosine then serves as a substrate for sphingosine kinase (SphK)-mediated phosphorylation to form mitogenic S1P. Hence, elevated ceramidase activity can reduce endogenous ceramide levels, thereby shifting the sphingolipid balance to a pro-survival state [13]. Five isoforms of ceramidase exist and are optimal in different pH environments: acid (ASAH1), neutral (ASAH2) and alkaline (ACER1-3). Acid ceramidase (hereafter referred to as AC), which is preferentially localized in the lysosome, is essential in embryogenesis and in tumor progression [14, 15]. AC is highly expressed in solid tumors isolated from prostate, melanoma, and breast cancers, as well as leukemia including T-cell large granular lymphocytic (LGL) leukemia [15–17]. Moreover, targeting AC induces programmed cell death (caspase-dependent or independent apoptosis) and increases sensitivity to cytotoxic agents [18–20].

Dysregulated apoptotic pathways are a common characteristic in cancers, including AML [21]. Anti-apoptotic myeloid cell leukemia sequence 1 (Mcl-1), a member of the Bcl-2 family, is overexpressed in AML [22]. Mcl-1 binds to and inhibits the activation of pro-apoptotic Bcl-2 family members, which prevents cytochrome *c* release and apoptosis [23]. Recent publications have shown that cancer cells can develop resistance to chemotherapeutic drugs and Bcl-2 inhibitors through Mcl-1 expression [24]. Furthermore, studies using *in vivo* AML models clearly demonstrated that Mcl-1 is essential in AML survival [25].

The present study explored the hypothesis that elevated AC plays a critical role in AML survival through sphingolipid dysregulation and Mcl-1 induction. We demonstrate that AC is upregulated in AML blasts and that AC inhibition with the ceramide analog LCL204 increased ceramide levels and induced apoptosis. AC inhibition also decreased Mcl-1 expression, uncovering a previously unknown regulation of Mcl-1. Taken together, these studies demonstrate for the first time that AC represents a novel and attractive target in AML.

## RESULTS

**Figure 1:**
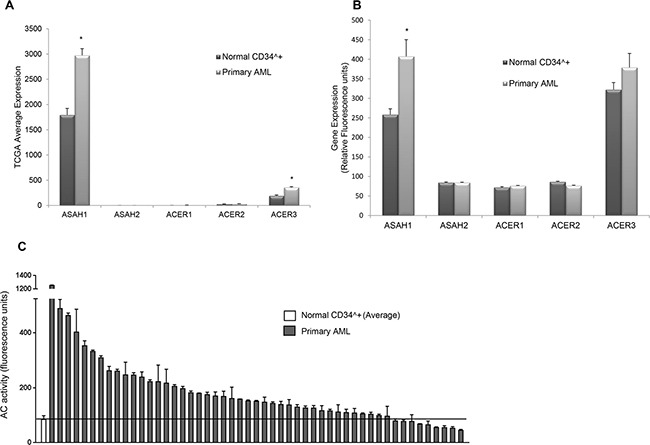
AC expression and activity are elevated in AML **A.** TCGA RNA-Seq gene expression data in 145 AML patients and 5 CD34^+^ normal BM samples for ceramidases ASAH1 (acid ceramidase), ASAH2 (neutral ceramidase), ACER1 (alkaline ceramidase 1), ACER2 (alkaline ceramidase 2) and ACER3 (alkaline ceramidase 3). Each bar represents mean relative fluorescence units, and error bars represent standard error of mean (SEM). *, *p*<0.05, indicates significant difference of AML patient cells compared to normal CD34^+^ cells (Wald test and Benjamini-Hochberg procedure). **B.** Microarray analysis of ASAH1, ASAH2, ACER1, ACER2 and ACER3 mRNA levels. The mRNA expression in AML patient cells (n =30) was compared to normal CD34^+^ bone marrow cells from healthy controls (n=8). Each bar represents mean relative fluorescence units, and error bars represent standard error of mean (SEM).*, *p*<0.05, indicates significant difference of AML patient cells compared to normal CD34^+^ cells (Wilcoxon rank-sum test). **C.** AC activity of normal CD34^+^ cells (n=12, far left, mean ± SEM) and primary AML patient samples (n=51) was measured using flourogenic substrate CB-12. AC activity is elevated in the vast majority of AML patient cells compared to normal controls (*p*=0.0016; Wilcoxon rank sum test). Solid line represents the normal mean.

We hypothesized that AC, which is elevated in several types of cancer, plays a critical role in AML [26–28]. Analysis of RNA-Seq data obtained from The Cancer Genome Atlas (TCGA, Figure 1A) showed that AML patient samples (n=145) have significantly higher (1.7-fold) AC expression compared to normal bone marrow samples (n=5) (FDR<0.05). This is unique only to AC, as other ceramidases were not highly or differentially expressed in AML patient samples or normal CD34^+^ bone marrow samples. Although ACER3 expression in TCGA AML patient samples is significant when compared to normal CD34^+^ bone marrow samples (FDR *p*<0.01), ACER3 expression is relatively low compared to ASAH1 expression. Independent gene expression microarray analysis (Figure 1B) of 30 AML patient samples and 8 normal CD34^+^ bone marrow samples verified that AC mRNA expression is significantly elevated (1.6-fold) in AML patients blasts compared to normal CD34^+^ bone marrow-derived samples (*p*<0.05, Mann-Whitney U- test). Next, we determined whether the observed increase in AC gene expression is associated with AC enzymatic activity. Samples were taken from 51 newly diagnosed AML patients and from enriched CD34^+^ cells from 12 normal donors and assayed for AC activity (Figure 1C). This screening indicated that 82% of AML patient samples tested have significantly higher AC activity compared to the mean AC activity of the normal CD34^+^ controls (*p*=0.0016; Wilcoxon rank sum test). Thus data from TCGA, microarray and AC enzymatic activity screening all show that AC is elevated in AML patient samples.

To determine whether high AC activity is relevant to therapeutic development, we sorted patients into high and low AC activity then correlated these measures with clinical outcomes. Patients who did not receive standard chemotherapy were excluded from these analyses. Patients with high AC activity had significantly lower overall survival (Supplementary Figure S1A, *p*=0.03, log-rank test) and significantly lower relapse-free survival than patients with lower AC activity (Supplementary Figure S1B, *p*=0.01, log-rank test). Patient distribution was relatively balanced among cytogenetic risk groups (Supplementary Figure S1C). This finding greatly supports AC's role in AML survival and suggests that it may be a clinically relevant biomarker of poor prognosis as well as a therapeutic target.

**Figure 2:**
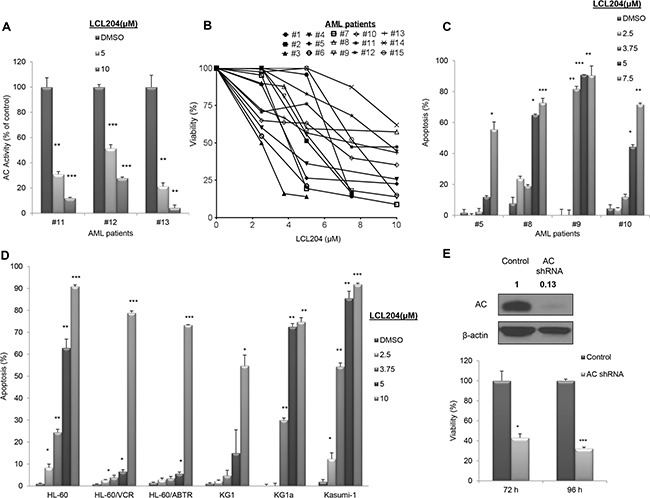
AC inhibition reduces viability and increases apoptosis in AML patient blasts and human AML cell lines **A-C.** AML patient samples were treated with LCL204 for 24 hours. (A) AC activity after LCL204 treatment in AML patient cells. (B) Viability and (C) apoptosis assays in LCL204-treated cells were performed as described in the Materials and Methods section. **D.** LCL204 treatment (24 hours) led to dose-dependent apoptosis induction in human AML cell lines. VCR: Vincristine resistant, ABTR: ABT-737 resistant. **E.** Viability was determined by MTS assay in HL-60/VCR cells electroporated with either control or AC shRNA pLKO.1 vectors. (Inset) Blot showing AC knockdown with AC shRNA pLKO.1 vector at 66 hours. *, *p*<0.05; **, *p*<0.005; ***, *p*<0.0005 versus control (Student's *t* test).

To establish whether this elevated AC expression and activity in primary AML cells is crucial to the survival of AML blasts, we treated patient cells with increasing doses of the AC inhibitor LCL204 [29] and measured the effect on AC activity, cell viability and apoptosis. Treatment of patient samples with LCL204 led to a dose-dependent, significant reduction in AC activity (Figure 2A). Samples from fifteen patients treated with LCL204 showed a dose-dependent decrease in cell viability (Figure 2B). Furthermore, 24-hour LCL204 treatment induced apoptosis in a dose- (Figure 2C) and time-dependent manner (Supplementary Figure S2) in patient samples. As AML is a heterogeneous disease, we screened six human AML cell lines with different molecular and cytogenetic abnormalities to determine the universality of sensitivity to LCL204. LCL204 induced dose-dependent apoptosis in all six AML cell lines tested, including the drug-resistant HL-60 variants HL-60/VCR and HL-60/ABTR (Figure 2D). Interestingly, AML cell lines were more sensitive to AC inhibition with LCL204 (EC_50_ 5.1 μM ± 0.2 SEM, n=7) than normal BM CD34^+^ cells (EC_50_ 6.0 μM, n=1) and normal mobilized PBMC CD34^+^ cells (EC_50_ 7.2 μM ± 0.4 SEM, n=4). AC activity was also significantly reduced with LCL204 treatment in HL-60/VCR starting at 1 μM (*p*<0.05, Student's *t*-test) (Supplementary Figure S3). A genetic approach was also utilized to further demonstrate the critical role of AC in AML survival. HL-60/VCR cells were electroporated with AC shRNA. AC expression was diminished at 66 hours (Figure 2E top), followed by decreased viability at 72 (*p*<0.05, Student's *t*-test) and 96 hours (*p*<0.0005, Student's *t*-test) post-transfection compared to control vector (Figure 2E bottom). These results indicate that elevated AC activity is crucial to the survival of primary AML blasts and human AML cell lines.

**Figure 3:**
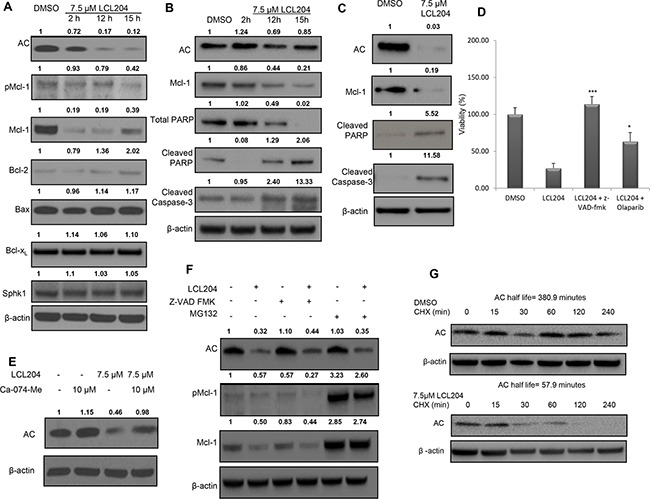
AC inhibition induces apoptosis, decreases pro-survival proteins and downregulates AC and Mcl-1 AML cell lines were treated with LCL204 and immunoblotted for apoptotic markers, as well as AC and Mcl-1. Band intensity ratios shown above the blots were calculated and normalized to β-actin with the first band in each blot as the reference band. **A.** HL-60/VCR cells treated with either DMSO (15 hours) or LCL204 (7.5 μM) for the indicated times. **B.** AML patient cells treated with either DMSO (15 hour) or LCL204 (7.5 μM) for the indicated time points (AML patient # 7) and **C.** for 18h (AML patient #16). **D.** HL-60/VCR cells were pre-treated with z-VAD fmk (25 μM) and olaparib (10 μM) for 1 hour before treating cells with LCL204 (7.5 μM) for 12 hours. Cells were analyzed by MTS assay and the data were normalized to DMSO control and reported as percent viability. **E.** HL-60/VCR cells were pre-treated with cathepsin B inhibitor Ca-074-Me (10 μM) for 1 hour, then treated with LCL204 (7.5 μM) for 5 hours. **F.** HL-60/VCR cells were co-treated with LCL204 (7.5 μM) and either pan-caspase inhibitor z-VAD fmk (10 μM) or proteasome inhibitor MG132 (5 μM) for 6 hours. **G.** HL-60/VCR cells were treated with DMSO or LCL204 (7.5 μM) for 1 hour, followed by 10 μg/ml cycloheximide (CHX) treatment for the indicated amount of time.

We further investigated the mechanism of LCL204-induced apoptosis to determine which apoptotic regulatory proteins were affected by LCL204 treatment. Extracts of HL-60/VCR cells treated with LCL204 were analyzed for Bcl-2 family expression levels (Figure 3A). LCL204 treatment dramatically decreased both AC and total Mcl-1 levels, first noted at 2 hours post-treatment. Phosphorylated Mcl-1 levels decreased at 15 hours post-treatment. However, little to no change was observed in other Bcl-2 family members (Bcl-2, Bcl-xL and Bax) with LCL204 treatment. Publications have shown that SphK1 can regulate Mcl-1 [30]; however, LCL204 treatment did not affect SphK1 expression (Figure 3A). To recapitulate these observations in patient samples, cells from two AML patients were treated with LCL204 (7.5 μM) in a time-dependent manner (Figure 3B) or a single dose for 18 hours (Figure 3C). AC inhibition induced markers of apoptosis with increased cleaved PARP and cleaved caspase-3 corresponding to the reduction in AC and Mcl-1 levels. Pre-treating cells with caspase inhibitor z-VAD-fmk (25 μM) or PARP inhibitor olaparib (10 μM) rescued the LCL204-mediated decrease in viability (Figure 3D), showing that caspase-3 and PARP are downstream effectors of LCL204-induced apoptosis. Both patient and cell line data demonstrated that LCL204 decreased AC and Mcl-1 levels and induced apoptosis associated with activation of caspase-3.

Because AC inhibition decreased both AC and Mcl-1 levels with only two hours of treatment, we probed further into the mechanism behind the rapid decrease in protein levels. Cathepsin B inhibition with Ca-074-Me prevented the loss of AC protein in LCL204-treated cells (Figure 3E), which is in agreement with existing evidence of a role for lysosomal proteases in this process [29]. Autophagy blockade with V-ATPase inhibitor Bafilomycin-A1 (Supplementary Figure S4) and caspase inhibition with z-VAD-fmk (Figure 3F) did not restore AC or Mcl-1 levels in LCL204-treated cells. However, proteasome inhibitor MG132 rescued LCL204-induced loss of Mcl-1 but did not rescue loss of AC (Figure 3F). Using cycloheximide to block protein synthesis, LCL204 reduced AC protein half-life from 380.9 minutes to just 57.9 minutes (Figure 3G). However, co-treatment of HL-60/VCR cells with LCL204 and actinomycin D demonstrated that LCL204 did not affect AC mRNA degradation rates (Supplementary Figure S5). Hence, our data show that short-term LCL204 treatment led to post-translational degradation of AC primarily through cathepsin B cleavage and Mcl-1 primarily through the proteasome.

**Figure 4:**
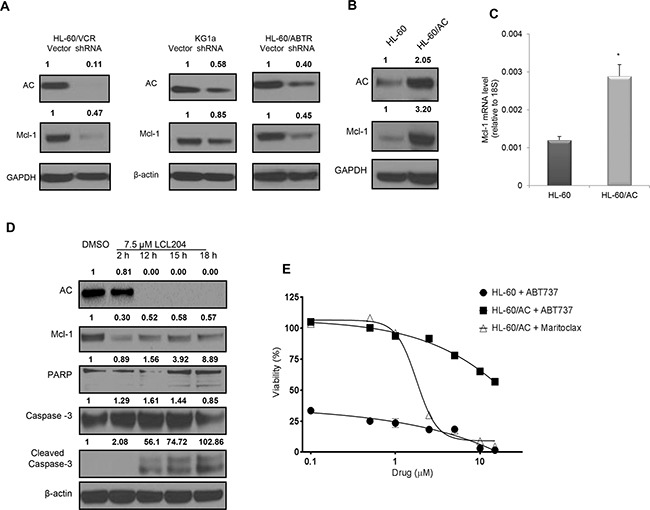
AC activity regulates expression of pro-survival Mcl-1 protein and confers resistance to Bcl-2 inhibition **A.** AC and Mcl-1 protein levels were detected by western blotting upon lentiviral-mediated AC knockdown in human HL-60/VCR (72 hours), HL-60/ABTR (60 hours) and KG1a cell lines (60 hours). Band intensity ratios shown above the blots were calculated and normalized to GAPDH or β-actin with the first band in each blot as the reference band. **B-C.** Increased Mcl-1 expression in HL-60 cells with AC overexpression (HL-60/AC) was quantified using (B) western blot and (C) qRT-PCR. **D.** HL-60/AC cells were treated with either DMSO (18 hours) or LCL204 (7.5 μM) for the indicated times and extracts were probed for the indicated proteins. **E.** HL-60 and HL-60/AC cells were treated with selective Mcl-1 and Bcl-2 inhibitors for 48 hours and analyzed for viability. *, *p*<0.05 versus control (Student's *t* test).

Several publications have found that Mcl-1 is essential for AML blast survival and functions through the sequestration of pro-apoptotic Bcl-2 family members [31, 32]. Transient knockdown of AC using lentiviral pLKO.1-AC shRNA in three AML cell lines also decreased Mcl-1 levels (Figure 4A), which verifies that LCL204-mediated effects were indeed related to loss of AC activity. We also demonstrated that LCL204 treatment for 24 hours decreased Mcl-1 mRNA levels dose-dependently (Supplementary Figure S6). To further study the relationship between AC and Mcl-1, we transduced HL-60 cells with pLOC-AC expression vector (referred hereafter as HL-60/AC). AC overexpression in HL-60 cells increased both Mcl-1 protein and mRNA expression, confirming that AC positively regulates Mcl-1 (Figure 4B and 4C). Furthermore, AC inhibition in HL-60/AC cells with LCL204 treatment also decreased both AC and Mcl-1 expression (Figure 4D). Like other AML cell lines tested, LCL204 treatment also induced apoptotic markers in HL-60/AC cells through caspase-3 and PARP cleavage in a time-dependent manner, starting at 12 hours post-treatment (Figure 4D). To determine whether Mcl-1 is the primary mediator of AC-stimulated survival, HL-60/AC cells were treated with Mcl-1 inhibitor maritoclax [32] and Bcl-2/Bcl-xL inhibitor ABT-737 [33] for 48 hours (Figure 4E). HL-60/AC cells were resistant to Bcl-2/Bcl-xL inhibition relative to parental HL-60 cells, but Mcl-1 inhibition reduced HL-60/AC cell viability to levels that matched HL-60 parental cells with ABT-737 treatment. Collectively, these results demonstrate that elevated AC enhanced AML blast survival through Mcl-1 upregulation.

**Figure 5:**
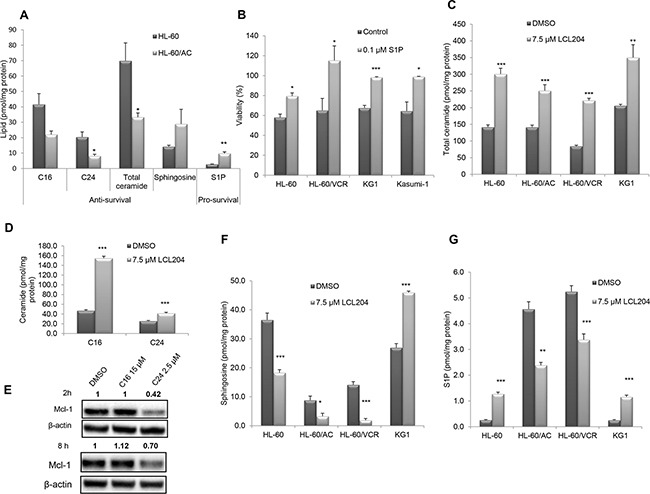
S1P promotes survival and AC activity regulates sphingolipid profiles in AML cell lines **A.** Enhanced survival phenotype upon AC overexpression. Pro-apoptotic lipid levels decreased while pro-survival levels were significantly elevated in HL-60/AC cells relative to parental cells. **B.** Exogenous S1P increases viability in AML cell lines. Cell lines were incubated for 24 hours with 0.1 μM S1P or vehicle control in Opti-MEM reduced-serum (50%) medium. Viability assay results are normalized to cells incubated with medium containing 10% serum. **C.** Total ceramide levels and **D.** C_16_ and C_24_ ceramides in HL-60/VCR increased with LCL204 treatment (7.5 μM) for 12 hours. **E.** Exogenous ceramide decreased Mcl-1 expression. HL-60/VCR cells were treated with DMSO, C_16_ ceramide (15 μM) or C_24_ ceramide (2.5 μM) for 2 hours and 8 hours, then Mcl-1 and β-actin levels were determined by western blotting. **F-G.** LCL204 treatment altered sphingosine and S1P levels in human AML cell lines. Cells were incubated with either DMSO or LCL204 (7.5 μM) for 12h and (F) sphingosine and (G) S1P levels were quantified by mass spectrometry as described in the Materials and Method section. *, *p*<0.05; **, *p*<0.005; ***, *p*<0.0005 versus control (Student's *t* test).

AC activity generates sphingosine, which in turn is phosphorylated by SphK to form S1P. As AC is elevated in AML samples and cell lines, we next determined how the sphingolipid content was altered in response to AC overexpression. Lipids from HL-60/AC cells exhibited decreased pro-apoptotic ceramide C_16_ and C_24_ and increased pro-survival lipid S1P (Figure 5A). Treatment of four human AML cell lines with S1P (0.1 μM) for 24 hours in Opti-MEM reduced serum medium significantly increased viability compared to the vehicle control cells, indicating that S1P enhances AML blast survival (Figure 5B). Conversely, AC inhibition in four AML cell lines with LCL204 treatment (7.5 μM) for 12 hours resulted in increased total ceramide content (Figure 5C). Among the ceramide species analyzed, C_16_ and C_24_ ceramides were significantly increased in HL-60/VCR cells (Figure 5D). Since LCL204 decreased Mcl-1 expression and increased ceramide levels, we treated HL-60/VCR cells with C_16_ and C_24_ ceramides for 2 and 8 hours to determine whether exogenous ceramide affects Mcl-1 protein levels. At both time points, C_24_ ceramide decreased Mcl-1 expression (Figure 5E), suggesting that LCL204 treatment stimulates Mcl-1 down-regulation through ceramide accumulation. LCL204 treatment for 12 hours also generally reduced sphingosine and S1P levels in AML cell lines (Figure 5F–5G). Although LCL204 did not affect SphK1 expression (Figure 2A), cells with higher levels of sphingosine had increased S1P levels with LCL204 treatment (Figure 5G). Thus AC inhibition increased ceramide levels while AC overexpression elevated pro-survival S1P. Collectively, the lipid analyses after LCL204 treatment show that the cytotoxic effects of LCL204 (Figure 2B–2D) correlated best with ceramide accumulation rather than S1P loss, thus indicating that elevated AC activity is an essential protective mechanism to deplete pro-apoptotic ceramide in AML.

**Figure 6:**
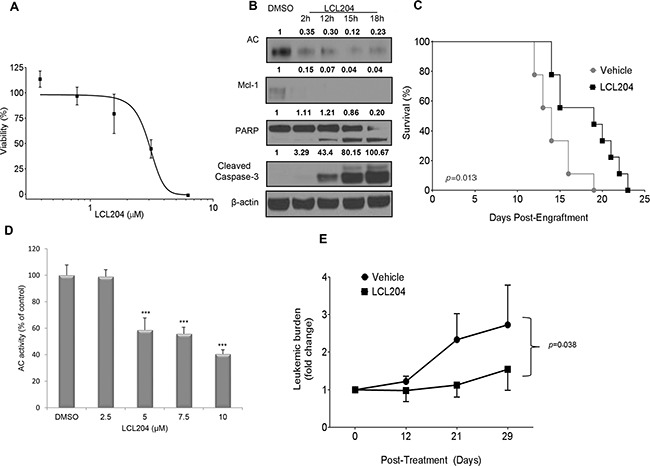
AC inhibition increases survival in a murine AML model and decreases leukemic blasts in a primary human AML xenograft model **A.** LCL204 treatment (48 hours) reduced viability of C1498 murine AML cells (EC_50_ = 3.0 μM). **B.** Western blot of C1498 lysates treated with either DMSO (18 hours) or LCL204 (7.5 μM) for the indicated times and probed with antibodies to apoptotic markers, AC and Mcl-1. LCL204 treatment induced apoptotic markers and decreased AC and Mcl-1 levels in C1498 cells. **C.** Median survival of C57BL/6 mice engrafted with syngeneic C1498 cells is significantly increased by LCL204 treatment (24 days) compared to vehicle-treated (PTD) controls (19 days; p=0.013, log-rank test). **D.** AC activity of human AML patient cells treated with LCL204 for 20 hours shows a significant dose-dependent decrease (***, p<0.0005 versus DMSO control (Student's t test)). **E.** NSG mice (n=12 per group) engrafted with primary human AML cells were treated with vehicle (PTD) or LCL204 (10 mg/kg) for 4 weeks. Leukemic burden (human CD45+/total human and mouse CD45^+^) was monitored by flow cytometry during the course of the treatment and is expressed as the fold change relative to the levels at initiation of treatment. LCL204 significantly decreased leukemic burden in leukemic NSG mice compared to vehicle-treated (PTD) mice (p=0.038, repeated-measure ANOVA).

Our *in vitro* experiments provided strong evidence that elevated AC activity induces survival in AML patient blasts and cell lines. We then utilized two established AML *in vivo* preclinical models to demonstrate that AC represents a novel target for AML treatment. C1498 is a syngeneic murine leukemic cell line of C57BL/6 origin [34]. The C1498 model is an aggressive leukemia which has previously been used to test the efficacy of chemotherapeutic agents [35, 36]. AC inhibition decreased C1498 cell viability in a dose-dependent manner with an apparent EC_50_ of 3 μM (Figure 6A). LCL204 treatment also decreased AC and Mcl-1 expression and induced apoptosis markers in C1498 cells (Figure 6B) as seen in human AML cell lines and patient samples (Figure 3). Maximum tolerated dose studies were conducted in C57BL/6 mice, and LCL204 showed minimal toxicity at up to 10 mg/kg (Supplementary Figure S7). C57BL/6 mice (n=9 per group) were engrafted with C1498 cells (1 x 10^6^) and treated with LCL204, starting at five days post-engraftment (5 mg/kg, administered three times/week, i. p.). Mice treated with this AC inhibitor had a significant increase in overall survival (24 days) compared to the vehicle control (19 days; *p*=0.013, log-rank test) (Figure 6C).

We then tested the preclinical efficacy of AC targeting in NOD scid gamma (NSG) mice engrafted with human patient-derived secondary AML cells. The NSG model was established to facilitate engraftment of human hematopoietic stem cells [37] and has been used extensively for studies involving human AML disease progression and preclinical therapeutics [38]. *In vitro* LCL204 treatment of this patient's cells for 20 hours (Figure 6D) significantly decreased AC activity (p<0.0005, Student's *t* test). NSG mice were engrafted with patient-derived cells and engraftment was confirmed before treatment began. Leukemic mice were then treated with either vehicle control or LCL204 (10 mg/kg, administered three times/week, i. v.) for 4 weeks. LCL204-treated mice showed a significantly reduced proportion of human leukemic cells (fold change in hCD45) compared to vehicle-treated mice (Figure 6E; p=0.038, repeated-measure ANOVA). Hence, our data from two AML *in vivo* models showed that targeting AC prolonged overall survival and decreased leukemic burden.

## DISCUSSION

Our findings identify AC as a novel target for AML therapeutic development. We show for the first time that AC is elevated in AML patient samples, and its activity is essential for survival and predictive of clinical outcome (Figure 1, 2 and Supplementary Figure S1). Current therapeutics exhibit limited efficacy in AML patients, especially in the poor prognostic group [5, 39]. The enzymes of ceramide metabolism are promising therapeutic targets in cancer [40]. AC catalyzes the breakdown of ceramide, and together with other ceramidases, provides the sole source of endogenous sphingosine [41]. Hence, high AC activity in AML blasts decreases cellular ceramide levels and increases sphingosine levels, which is ultimately converted to S1P by sphingosine kinases. AC's dual role in regulating both ceramide and sphingosine levels increases its importance in sphingolipid dysregulation. Studies by our group and others indicate that there are a multitude of perturbations of sphingolipid metabolism in AML, some of which create alternative mechanisms of S1P elevation or ceramide detoxification. We see that AC activity is elevated in the vast majority of AML samples versus control and that AC inhibition shows some degree of efficacy in all cell lines and patient samples tested thus far. Although identical analyses in the TCGA AML cohort showed no prognostic significance of AC mRNA expression levels (data not shown), our patient cohort showed significant association between high AC activity and shorter overall survival as well as relapse-free survival, thus establishing AC as a key biochemical target in AML.

Elevated AC can stimulate AML survival, and our data demonstrate that inhibiting AC reduces the viability of AML cells. LCL204 treatment decreased viability by inducing apoptosis in AML patient blasts and cell lines through the activation of caspase-3 and cleaved PARP. These effects were preceded by rapid post-translational degradation of AC and Mcl-1. LCL204-mediated release of lysosomal cathepsin B and degradation of AC has been previously reported [29]. LCL204 treatment led to selective loss of Mcl-1 while other Bcl-2 family members were unaffected. This degradation was mediated via the proteasome and loss of Mcl-1 was recapitulated by exogenous ceramide supplementation as well as genetic knockdown of AC expression. Overall, our findings showed an increased dependence of AML blasts on AC-mediated ceramide detoxification and pro-survival signals.

Our findings also clearly show that AC inhibition via LCL204 significantly increases ceramide levels. LCL204 treatment increased C_16_, C_24_ ceramides and total ceramide in all AML cell lines within 12 hours. This strongly suggests that ceramide accumulation impairs AML blast survival and that the levels are kept low through elevated AC activity. LCL204 also decreased sphingosine and S1P levels in several AML cell lines. Cell lines with higher levels of sphingosine tend to exhibit increased S1P even after LCL204 treatment, which is possible since this conversion is downstream of AC. Overall, our results are consistent with LCL204-mediated AC degradation following release of cathepsin B. The loss of AC activity prevents ceramide conversion to sphingosine and leads to rapid ceramide accumulation that, together with the loss of pro-survival Mcl-1, results in robust induction of apoptosis. Genetic manipulation through AC knockdown also reduces viability in AML cell lines and decreases Mcl-1 expression, showing that LCL204 acts primarily through AC inhibition rather than off-target effects. Conversely, AC overexpression increases pro-survival lipids by decreasing total ceramide levels and increasing S1P levels. Our results reflect on the heterogeneity of AML by showing that different patients or AML cell lines exhibit varying response to AC inhibition. Taken together, we provide strong evidence that AC activity enhances AML blast survival, and demonstrate for the first time that targeting AC reverses the aberrant AML lipid profile of low ceramide levels and elevated S1P, which ultimately leads to cell death.

Our data demonstrate that AC regulates Mcl-1 to induce survival in AML. Mcl-1 has been shown to prolong blast survival in AML mouse models and is highly expressed in AML blasts [25, 42]. Here, we report decreased Mcl-1 protein expression with AC inhibition or knockdown as well as loss of Mcl-1 with ceramide treatment. Several publications have recently shown that targeting Bcl-2 and Bcl-xL using Bcl-2 family inhibitors decreased survival of AML patient cells and cell lines [43–45]. Our data show that LCL204 did not affect other Bcl-2 family members, and we did not uncover any direct relationship between AC and other Bcl-2 family members. The mechanism by which elevated AC activity affects AML survival through Mcl-1 expression could be explained through the “sphingolipid rheostat” [8]. Ceramide can reduce Mcl-1 expression or inactivate Mcl-1 [46–49]. Our data showed that exogenous C_24_ ceramide reduced Mcl-1 expression to a greater extent than pro-apoptotic C_16_ ceramide, which suggest a role for long chain ceramides in apoptotic regulation. In addition, previous studies indicate that Mcl-1 binding to BAK modulates the ability of BAK to regulate ceramide synthase activity, which may represent yet another interplay between sphingolipid metabolism and apoptotic pathways [50, 51]. The reduced ceramide levels associated with AC overexpression corresponded with higher S1P levels. S1P has been shown to act as a co-factor for E3 ubiquitin ligase TRAF2, which leads to the activation of NF-κB, a direct and indirect regulator of Mcl-1 [52–55]. Further studies are needed to thoroughly explore mechanisms underlying AC's regulation of Mcl-1 and potential effects of AC overexpression on mRNA stability, protein translation or post-translational events.

Targeting AC increases overall survival of C1498 leukemic mice and decreases leukemic burden in patient-derived leukemic NSG mice. Furthermore, our murine *in vivo* and human *in vitro* data also suggest a therapeutic window for LCL204 as AML cells exhibit higher sensitivity to LCL204 than normal counterparts as minimal toxicity was observed. This is an exciting finding for AML therapeutics. Future experiments will extend these findings to determine effects on overall survival with multiple AML patient samples of varying clinical and molecular subtypes in the NSG mouse model.

Our data clearly indicate that AC expression and activity are elevated in AML, which dysregulates the sphingolipid rheostat, and demonstrate that AC is a novel therapeutic target in AML. Elevated AC activity also upregulates Mcl-1 to induce survival in AML blasts, and our data show that targeting AC decreased Mcl-1 levels as well as increased ceramide levels in AML cells to induce cell death. Therefore, future clinically approved AC inhibitors may be utilized in synergy with existing treatment to enhance therapeutic response in AML patients. As AML is a heterogeneous group of hematological malignancies, further delineation of the molecular and cytogenetic classification(s) of AML patients that exhibit elevated AC activity could identify subgroups that may benefit most from AC-targeted treatments.

## MATERIALS AND METHODS

### Patient samples, cell lines, and inhibitors

All AML patient samples with 20% or greater blast count were collected with signed informed consent according to a protocol approved by the Institutional Review Board of the Milton S. Hershey Medical Center. Patients were categorized into cytogenetic risk groups based on published guidelines [56]. Human G-CSF mobilized PBMC samples from healthy donors were obtained from the Blood Bank (Milton S. Hershey Medical Center). PBMCs were enriched using the Ficoll-Paque gradient separation method (Pharmacia Biotech, Piscataway, NJ). CD34^+^ cells were isolated from PBMCs with a CD34^+^ microbead kit (Miltenyi Biotec, Cambridge, MA). CD34^+^ bone marrow cells from normal donors were purchased from All Cells, LLC. (Emeryville, CA).

HL-60, KG-1, KG-1a, C1498 and Kasumi-1 were purchased from American Type Culture Collection (ATCC, Manassas, VA). The ABT-737-resistant HL-60 (HL-60/ABTR) cells were generated as previously described [32]. Vincristine-resistant HL-60/VCR cells were selected as previously described [57]. Kasumi-1 was grown in RPMI-1640 (Invitrogen, Carlsbad, CA) supplemented with 20% FBS from Hyclone (Thermo Fisher Scientific, Waltham, MA). KG1 and KG1a were grown in IMDM supplemented with 20% FBS. All other cell lines were grown in RPMI-1640 supplemented with 10% FBS. In addition, HL-60/VCR cells were maintained in medium containing 2 μg/ml vincristine. All cells were grown in a 37°C humidified, 5% CO_2_ atmosphere incubator. All cell lines were authenticated using short tandem repeat DNA profiling (Genetica DNA laboratories) at the completion of studies.

LCL204 [(1R,2R) 2-(N-tetradecylamino)-1-(4-NO2)-phenyl- 1,3-dihydroxy-propane HCl] was synthesized as previously described [29]. CB-12 was kindly provided by Dr. Gemma Fabrias (IQAC-CSIC, Barcelona, Spain) [58]. Marinopyrrole A (maritoclax) was synthesized as previously described [32, 59]. ABT-737, MG132, z-VAD-fmk, actinomycin D and Ca-074-Me were purchased from Selleckchem (Houston, TX). Cycloheximide was purchased from Sigma-Aldrich (St. Louis, MO).

### AC activity

AC activity was measured by the accumulation of the fluorescent umbeliferone product from the substrate CB-12 as previously described [58]. Cells (2 x 10^4^) were incubated in RPMI-10% FBS containing 16 μM of CB-12 for 3 hours at 37°C. The reaction was stopped by adding 50 μl of 100% methanol and 100 μl of 2.5 mg/ml sodium periodate in 100 mM glycine, pH 10.6. Cells were incubated at 37°C for an additional 2 hours. Fluorescence was measured in the UV range using a Synergy HT plate reader (Biotek, Winooski, VT) and normalized to blank reading of RPMI-10% FBS and fluorogenic substrate. AC activity is presented as mean fluorescence unit per 20,000 cells.

### Ceramidase gene expression: RNA-Seq and microarray

RNA-Seq: average gene expression from TCGA AML patients and 5 normal CD34^+^ BM controls was expressed as a mathematical mean and standard error of normalized read counts as provided by DESeq2 [60]. Significance for differential expression was determined by DESeq2 using the Wald test (default significance test), and adjusted for multiple hypothesis testing using Benjamini-Hochberg FDR [61] across all genes tested.

Microarray: Total RNA was harvested from 30 AML patient samples and 8 CD34^+^ bone marrow samples from healthy donors (All Cells) using TRIzoL Reagent (Invitrogen) according to the manufacturer's instructions. Gene expression analysis was carried out using Illumina Human HT-12 V.3 Expression BeadChip (Illumina, San Diego, CA). The beadarray expression data were imported and quantile-normalized using the R statistical computing environment and the beadarray Bioconductor package. Hybridization data and parameter information can be accessed in the Gene Expression Omnibus (GEO) database (http://www.ncbi.nlm.nih.gov/geo). The GEO platform accession number is GSE65409. The Illumina CHP and CEL file accessions are GSM1595702-GSM1595739. Expression data for ASAH1, ASAH2 and ASAH3 probes were averaged and graphed as relative fluorescence units.

### *In vitro* apoptosis assay

Cells 2.5 x 10^5^ were treated with DMSO (final concentration less than 1% of total volume) or LCL204 at various doses and time points. Cellular apoptosis was quantified by flow cytometry using Annexin V-FITC (BD Pharmingen, San Diego, CA) following the manufacturer's protocol. 7-amino-actinomycin D (7-AAD; BD Pharmingen) was used to determine dead cells. Flow cytometry was done on the BD FacsCalibur. The percentage apoptosis was calculated based on the Annexin-V-FITC positive population.

### *In vitro* viability assay

Cells 2 x 10^4^ were plated in a 96-well plate and incubated with drug or DMSO control (less than 1% of final volume) for the indicated time points. For experiments using z-VAD-fmk and olaparib, cells were pre-treated with the inhibitors for 1 hour before LCL204 or DMSO treatment. All viability assays were measured using CellTiter 96 Aqueous One Solution assay kit (Promega, Madison, WI) according to the manufacturer's protocol. The absorbance of the formazan product at 490 nM was determined using a BioTek Synergy HT plate reader.

For S1P-treatment assays, cells were cultured in Opti-MEM Reduced Serum Medium (with 50% reduced serum supplementation) (Invitrogen) for 30 hours. S1P (Cayman Chemicals, Ann Arbor, MI) was first dissolved in methanol to make a 1 mM stock then diluted in BSA solution (32 mg/ml) to 125 μM for MTS assays. Methanol-BSA solution (final concentration 1.04% methanol) was used as vehicle control for the experiment. Absorbances were normalized to cells incubated with medium containing 10% serum and recorded as percentage viability.

### Real-time quantitative RT-PCR

Real-time quantitative reverse transcription polymerase chain reaction (qRT-PCR) was performed using primer sets specific for human ASAH1 and MCL1, with Beta-2- microglobulin (B2M) or 18S as internal standards in an ABI PRISM 7900 sequence detector. Total RNA was harvested from cells using TRIzoL Reagent (Invitrogen) following the manufacturer's protocols. cDNA was synthesized using random hexamers and MMLV reverse transcription reagent (Invitrogen). Amplification of the cDNA was performed in triplicate using Quantitect SYBR Green PCR kit (Qiagen) following the manufacturer's instructions. Primer sequences are as follows: ASAH1 sense 5′-TGT GGA TAG GGT TCC TCA CTA GA-3′, antisense 5′-TTG TGT ATA CGG TGA GCT TGT TG-3′; MCL1 sense 5′-CAA GGG AAG CTT TTC CTC TC-3′, antisense 5′-CAT GGA AAC CAA GCC AAA GT-3′; B2M sense 5′-TGC TGT CTC CAT GTT TGA TGT ATC T-3′, antisense 5′-TCT CTG CTC CCC ACC TCT AAG T-3′; 18S sense 5′-GTA ACC CGT TGA ACC CCA TT-3′, antisense 5′-CCA TCC AAT CGG TAG TAG CG-3′. All primers were supplied by IDTDNA (Coralville, IA).

### Overexpression and knockdown of AC via lentiviral transduction and transfection

AC-expressing plasmid pLOC-AC was purchased from Open Biosystems (Thermo Scientific) and was transfected into HEK293-FT cells with lentiviral packaging plasmids (Invitrogen). Viral particles were added to cells every 12 hours for 3 days with 6 μg/ml polybrene. Transduced cells were selected with 6 μg/ml of Blasticidin S for 2 weeks. For AC knockdown, plasmid pLKO.1 containing AC shRNA sequences (Mission; Sigma-Aldrich) was used following the same protocol. Lysates were harvested 60 to 90 hours post-transduction. Neon^®^ transfection system (Invitrogen) was used to electroporate pLKO.1 plasmids according to manufacturer's protocols.

### Western blot analysis

Cells were lysed using RIPA buffer containing phosphatase inhibitor cocktail 2 and protease inhibitor P8340 according to the manufacturer's protocol (Sigma), resolved on 4-12% SDS-PAGE gels and transferred onto PVDF membranes (Millipore, Billerica, MA). Primary antibodies used in this study are as follows: AC (BD Biosciences), AC (Aviva Systems Biology; San Diego, CA), anti-murine Mcl-1 (Rockland; Gilbertsville, PA), and from Cell Signaling (Danvers, MA): Bcl-2 (#4223), Mcl-1 (#5453), phospho-Mcl-1 (#4579), Bax (#5023), Bcl-xL (#2764), PARP (#9532), SPHK1 (#12071), Caspase-3 (#9662), GAPDH (#2118) and Beta-actin (#3700). For secondary antibodies, HRP-conjugated goat anti-mouse or goat anti-rabbit IgG (Cell Signaling) was used and Pierce Enhanced Chemiluminescence (Thermo Scientific) was applied to blots according to manufacturer's protocol. Image Processing and Analysis in Java (Image J) software was used to measure band density. Each band density was normalized to GAPDH or β-actin with the first band in each blot as the reference band.

### Lipid extraction and analysis by electrospray ionization-tandem mass spectrometry

Sphingolipids were analyzed by LC/ESI-MS/MS based on the method described with some modifications as described below [62]. Lipids were extracted from cell pellets (equivalent to 600 μg to 1 mg of protein depending on the experiment) using an azeotrophic mix of isopropanol:water:ethyl acetate (3:1:6; v:v:v). Internal standards (50 pmol of d17 long-chain bases and C12 acylated sphingolipids) were added to samples at the onset of the extraction procedure. Extracts were separated on an Agilent 1100 HPLC system. Mobile phases were as described [62], but with 0.2% formic acid on a Phenomenex Luna C8 (3 μm) 2.1 mm ID × 5 cm column maintained at 60°C for separation of the sphingoid bases and 1-phosphates or a Supelco 2.1 mm ID x 5 cm NH2 column for acylated sphingolipids. The eluate was analyzed with an inline ABI 4000 Q Trap (SCIEX, Framingham, MA) mass spectrometer equipped with a turbo ion spray source. Mass spectrometry settings were set to compensate for differences in ionization efficiency of different acyl chain lengths. The peak areas for the different sphingolipid subspecies were compared with that of the internal standards with 13C isotopic signals compensated for where necessary. All data reported are based on monoisotopic mass and are represented as pmol/mg protein.

### Exogenous ceramide assay

C_16_ and C_24_ (Avanti) were dissolved in 100% Methanol and then diluted 98:2 (v/v) into dodecane. HL-60/VCR cells were plated at 1 x 10^6^ cells per ml in RPMI medium supplemented with 10% FBS. The cells were treated with ceramide for 2 hours and 8 hours, after which the cells were harvested for western blot.

### C57BL/6 and NSG mouse *in vivo* models

To generate the *in vivo* C1498 murine AML model, 1 x 10^6^ C1498 cells were injected retro-orbitally into syngeneic eight-week-old female C57BL/6J mice (Jackson Labs, Bar Harbor, ME) [63]. LCL204 was dissolved in 30% v/v propylene glycol, 5% v/v Tween 80 and 65% of 5% w/v dextrose in water (PTD). Five days after C1498 engraftment, 5 mg/kg of LCL204 or PTD vehicle was injected i.p. every 2 days for a total of 10 injections. The amount of LCL204 administered was based upon two maximum tolerated dose studies and the regimen was based on a previously published method [28]. Mice with 20% body weight loss were considered to have significant leukemic burden and were euthanized.

Twenty-four female NSG (NOD.Cg-*Prkdc^scid^*Il2rg*^tm1Wjl^*/SzJ) mice (6-8 week-old, Jackson Laboratory) were sub-lethally irradiated (200 cGy) the day before intravenous injection of 5.5 x 10^5^ patient-derived secondary AML cells. Five months following injection, AML engraftment was confirmed and the mice were randomly divided into two groups and treated with 10 mg/kg LCL-204 i.v. or vehicle control PTD thrice a week for 4 weeks. Blood was collected to monitor the tumor burden at different time points. CD45-FITC (BD 555482), CD33-PE (BD 555450), CD19-APC H7 (BD 560727), CD3-PE Cy7 (BD 557851), HLA DR-PerCP Cy5.5 (BD 560652), CD117-BV421 (BD 562434), and mCD45-BV650 (BD 563410) were used to determine leukemia burden by flow cytometry (LSR-II, BD Biosciences). The leukemia burden was calculated as number of human CD45 positive cells/ (total number of human CD45 positive cells + mouse CD45 positive cells) x 100. All animal studies were conducted in accordance with the guidelines approved by the Institutional Animal Care and Use Committee at The Pennsylvania State University, College of Medicine.

### Statistical analyses and experimental design

Statistical analyses between two treatment groups were performed with Student's *t*-test. Mann-Whitney U- test was used in the microarray analysis and Wilcoxon rank sum test was used in the AC activity screening analysis. Other statistical tests used were described alongside individual experiments in the materials and methods, figure legends and results sections. Each graph was presented as an average of three replicates and error bars represent standard error of mean (SEM). For *in vitro* assays using cell lines, results were from two or more independent experiments, unless otherwise noted. Due to the limitation of primary samples, data from primary patient and normal donor samples were from one independent experiment.

Supplementary information is available at *Oncotarget*'*s* website.

## SUPPLEMENTARY FIGURES


