# Supplementary material for: Acid ceramidase is upregulated in AML and represents a novel therapeutic target
Source: Oncotarget. 2016 Nov 4;7(50):83208–22. doi: 10.18632/oncotarget.13079 (PMC5347763; doi:10.18632/oncotarget.13079)
Supplement: Supplementary file 1 [file oncotarget-07-83208-s001.pdf]

Acid ceramidase is upregulated in AML and represents a novel therapeutic target

SUPPLEMENTARY FIGURES

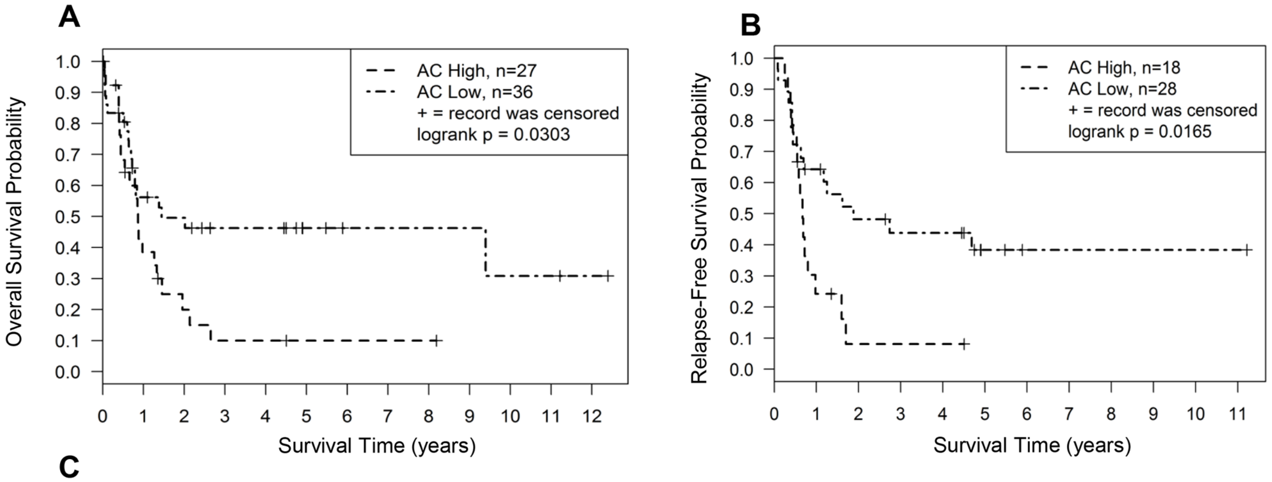

C

AC activity	Total cases	Good	Intermediate	Poor	Indeterminate/ not done
Low	36	3	18	6	9
High	27	2	12	7	6

Supplementary Figure S1: High AC activity correlated with lower overall survival and lower relapse-free survival in AML patients. AC activity values were log-transformed and centered according to the mean of the transformed values. The values were dichotomized into high/low categories according to whether they are larger than the mean. Patients were filtered to include only those that received standard chemotherapy “7+3”. The main clinical outcomes used were **A.** overall survival (OS) and **B.** relapse-free survival (RFS). Kaplan-Meier plots and log-rank tests were used to examine the correlation between AC level and OS/RFS. The analysis of RFS was only done for patients who had complete remission (CR) or complete remission with incomplete hematologic recovery (CRi) after primary therapy. All analyses were done using statistical software SAS version 9.4 (SAS Institute, Cary, NC, USA) and R programming language version 3.2.5 (R Foundations). All tests were two-sided and the statistical significance level used was 0.05. **C.** Distribution by cytogenetic risk status of patients used in Supplementary Figure S1A with high or low AC activity, as outlined by Slovak, M.L., et. al., Karyotypic analysis predicts outcome of preremission and postremission therapy in adult acute myeloid leukemia: a Southwest Oncology Group/ Eastern Cooperative Oncology Group study, Blood, 2000.

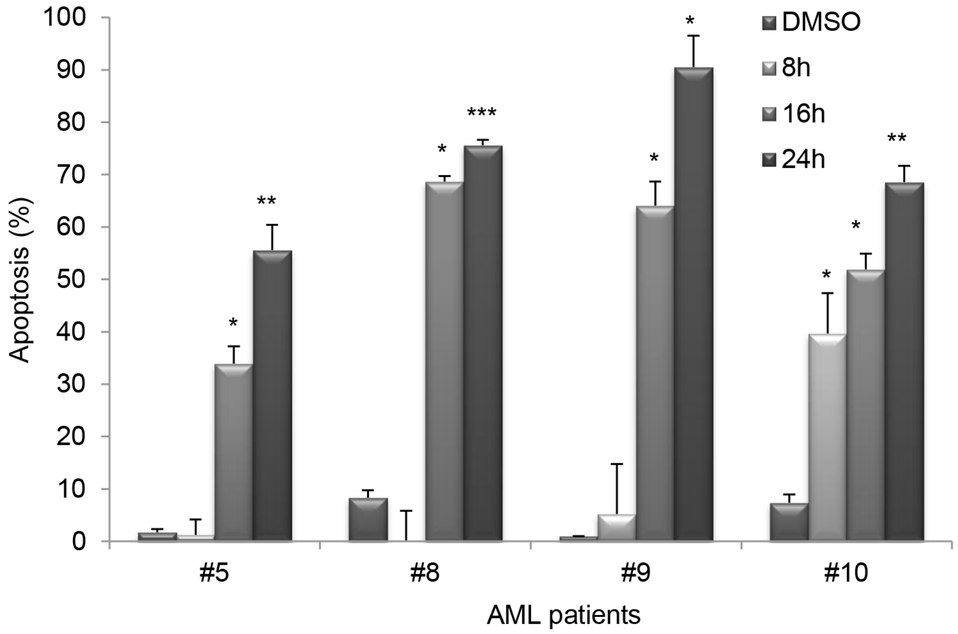

Supplementary Figure S2: LCL204 induced apoptosis in primary AML samples in a time dependent manner. AML patients samples were treated with DMSO for 24h or LCL204 (7.5 µM) for the indicated amount of time. Apoptosis assay was conducted as described in Materials and Methods section. *, $p<0.05$; **, $p<0.005$ versus control (Student's t test).

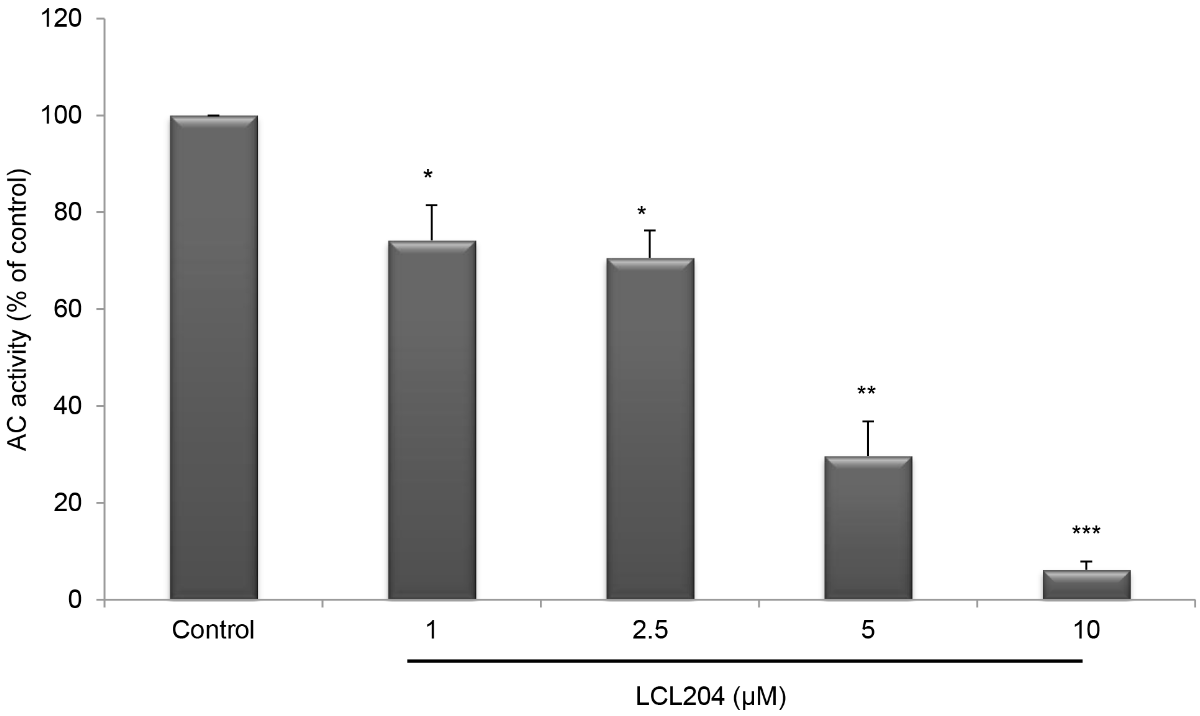

Supplementary Figure S3: AC activity decreased with LCL204 treatment. HL-60/VCR cells were treated with DMSO or LCL204 for 24 h. AC activity was quantified as described in Materials and Methods and is expressed relative to vehicle control. *, $p<0.05$; **, $p<0.005$; ***, $p<0.0005$ versus control (Student's t test).

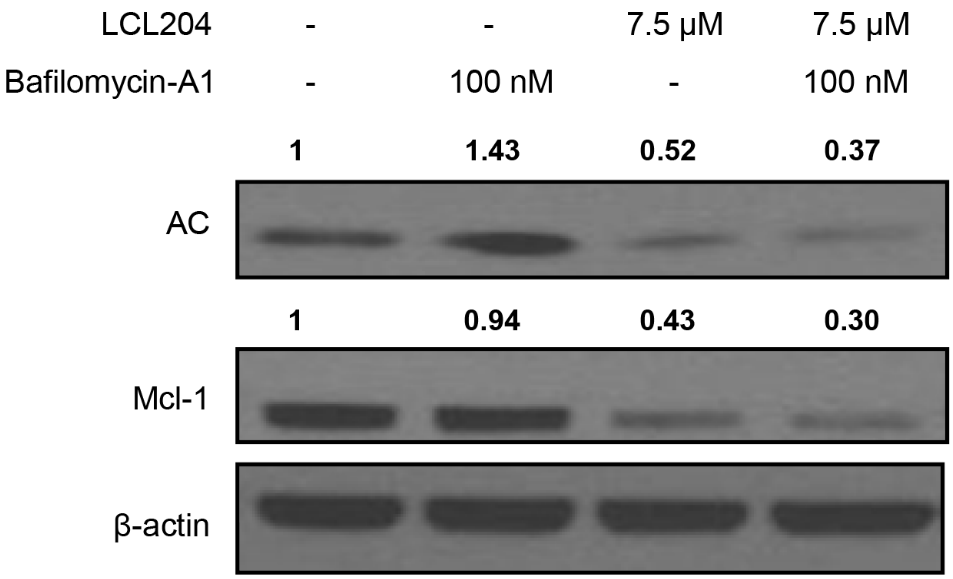

Supplementary Figure S4: Autophagy blockade did not rescue AC and Mcl-1 from LCL204-induced degradation. HL-60/VCR cells were pre-treated with Bafilomycin-A1 for 1h, followed by 5h treatment with LCL204 or DMSO vehicle.

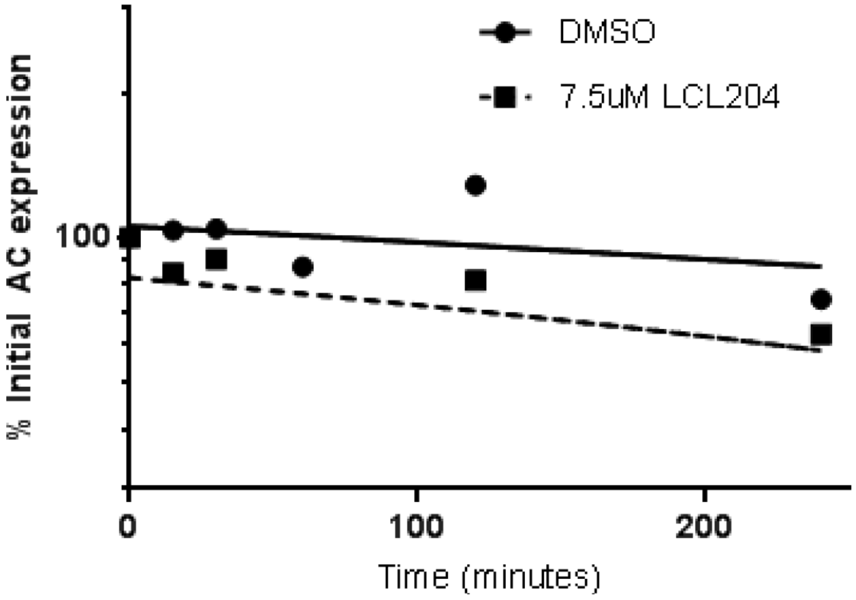

Supplementary Figure S5: LCL204 did not affect AC mRNA degradation. HL-60/VCR cells were pre-treated with 7.5 µM LCL204 or DMSO for 1h, followed by treatment with 5ug/ml of actinomycin D. Cells were collected at various time points and RNA was extracted. qRT-PCR assay was conducted and AC mRNA expression was calculated and plotted as % initial expression.

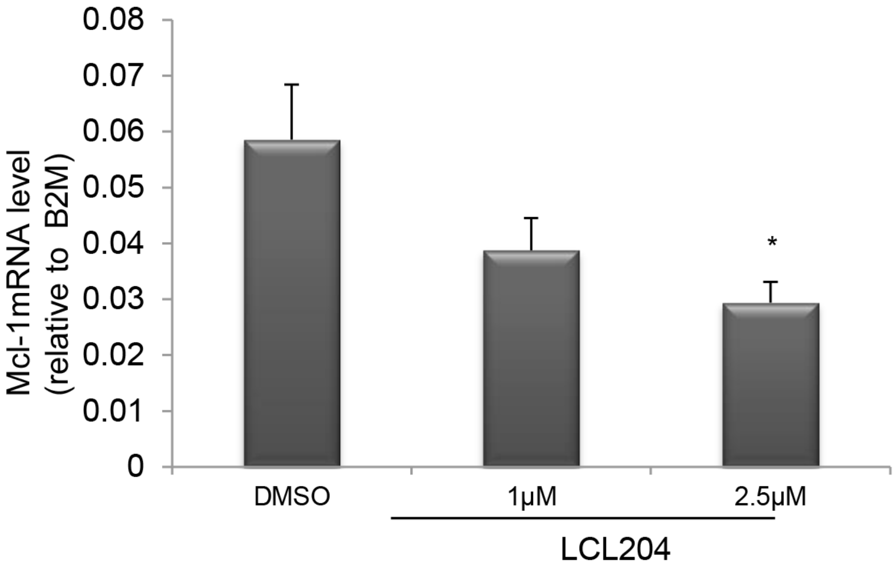

Supplementary Figure S6: LCL204 decreased Mcl-1 mRNA expression. HL-60/VCR cells were treated with DMSO or LCL204 for 24 hours. Mcl-1 mRNA was measured using qRT-PCR and normalized to B2M expression. *, p<0.05 versus control (Student's *t* test).

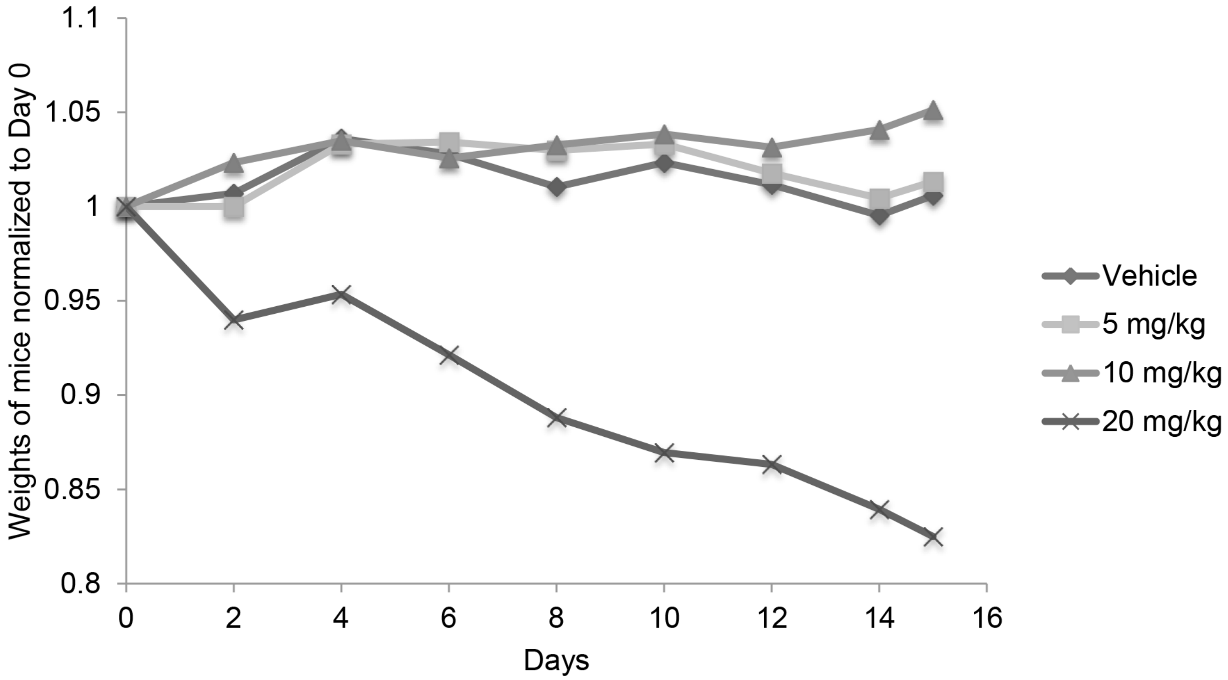

Supplementary Figure S7: LCL204 treatment did not induce overt toxicity and had minimal effect on body weights of C57BL/6 mice. C57BL/6 mice were injected with LCL204 every 2 days starting on Day 0 (7 injections total). Mice were sacrificed on Day 15. Data were normalized to weight measurements on Day 0.
